# Uremia does not affect neointima formation in mice

**DOI:** 10.1038/s41598-017-06816-6

**Published:** 2017-07-26

**Authors:** Annemarie Aarup, Carsten H. Nielsen, Line S. Bisgaard, Ilze Bot, Henrik H. El-Ali, Andreas Kjaer, Lars B. Nielsen, Tanja X. Pedersen

**Affiliations:** 1 Department of Biomedical Sciences, University of Copenhagen, Copenhagen, Denmark; 2 Department of Clinical Physiology, Nuclear Medicine & PET and Cluster for Molecular Imaging, Rigshospitalet, University of Copenhagen, Copenhagen, Denmark; 3 Division of Biopharmaceutics, Leiden Academic Centre for Drug Research, Leiden University, Leiden, The Netherlands; 4 Copenhagen University Hospital, Rigshospitalet, Department of Clinical Biochemistry, Copenhagen, Denmark

## Abstract

Atherosclerotic cardiovascular disease is a major complication of chronic kidney disease (CKD). CKD leads to uremia, which modulates the phenotype of aortic smooth muscle cells (SMCs). Phenotypic modulation of SMCs plays a key role in accelerating atherosclerosis. We investigated the hypothesis that uremia potentiates neointima formation in response to vascular injury in mice. Carotid wire injury was performed on C57BL/6 wt and apolipoprotein E knockout (*Apoe* ^−/−^) mice two weeks after induction of uremia by 5/6 nephrectomy. Wire injury led to neointima formation and downregulation of genes encoding classical SMC markers (i.e., myocardin, α-smooth muscle actin, SM22-alpha, and smooth muscle myosin heavy chain) in both wt and *Apoe* ^−/−^ mice. Contrary to our expectations, uremia did not potentiate neointima formation, nor did it affect intimal lesion composition as judged from magnetic resonance imaging and histological analyses. Also, there was no effect of uremia on SMC marker gene expression in the injured carotid arteries, suggesting that there may be different effects of uremia on SMCs in different vascular beds. In conclusion, uremia does not accelerate neointima formation in response to wire injury of the carotid artery in mice.

## Introduction

Chronic kidney disease (CKD) is characterized by loss of kidney function and leads to accumulation of waste products in the blood, i.e. uremia^1, 2^. The number of patients with decreased kidney function is rising worldwide, partly caused by the increased prevalence of diabetes^3, 4^. Cardiovascular disease is a major complication for CKD patients and accounts for more than 50% of deaths in dialysis-dependent patients^5, 6^. This is in part due to accelerated atherosclerosis^7^. Even slightly reduced renal function, which affects more than 10% of the population, is associated with increased atherosclerosis and cardiovascular mortality^8–12^. The association between CKD and cardiovascular disease cannot solely be explained by traditional cardiovascular risk factors^8, 13^. Decreased kidney function is an independent risk factor for development of atherosclerotic lesions^7^, and statin treatment has reduced effects in patients with advanced CKD^14^. Thus, studies investigating the mechanisms behind the accelerated atherosclerosis in uremia are warranted.

To enable such analyses, a mouse model in which moderate uremia accelerates atherosclerosis has been established^15–17^. Using this model, it was shown that uremic atherosclerosis is associated with degenerative changes in the aortic tunica media of hyperlipidemic apolipoprotein E knockout (*Apoe* ^−/−^) mice^18^.

In the healthy artery, the tunica media largely consists of contractile vascular smooth muscle cells (SMCs). SMCs possess enormous plasticity, and play an important role in the development of classical atherosclerosis. Upon vascular injury, e.g. atherosclerosis or carotid wire injury, SMCs undergo phenotypic modulation from a quiescent “contractile” state to a more dedifferentiated “synthetic” state. This phenotypic shift is accompanied by reduced expression of SMC marker proteins, such as the transcription co-factor myocardin (MYOCD), smooth muscle alpha actin (ACTA2), smooth muscle 22-alpha (TAGLN) and smooth muscle myosin heavy chain (MYH11)^19, 20^. In the synthetic state, SMCs become proliferative, migratory, lose contractility, and increase the synthesis of extracellular matrix proteins^21^. SMCs display immense plasticity, not only within the SMC phenotypes, but also with the ability to transdifferentiate into e.g. macrophage-like cells^22, 23^. Cholesterol uptake by SMCs induces downregulation of classical SMC markers and a more macrophage-like phenotype^22^. Recent lineage –tracing studies have shown that more than 80% of cells with SMC origin are not ACTA2 positive, and that 30% of cells classified as macrophages originate from SMCs in mouse atherosclerotic lesions^24^. Hence, although SMC modulation is important for vessel repair in response to injury, it also contributes substantially to development of diseases of the vascular wall. This suggests that SMCs may play a more pro-atherogenic role than previously believed.

We have recently observed that uremia leads to downregulation of contractile SMC marker genes both in areas with and without atherosclerotic lesions^25^. This downregulation was consistent in normocholesterolemic wild type mice, in which the contractility of the uremic aorta was also reduced. Furthermore, serum from CKD patients decreases mRNA expression of contractile genes, and increases proliferation of aortic SMCs^26^. Together, these observations indicate that uremia may directly affect phenotypic modulation of SMCs. Given the emerging evidence that SMCs play important roles in vascular injury, we hypothesized that uremia-mediated phenotypic modulation of SMCs plays a key role in uremic vasculopathy. To test this hypothesis, we performed carotid wire injury in uremic wild type and *Apoe* ^−/−^ mice.

## Results

### Uremia does not affect neointima formation in C57BL/6 mice

**Figure 1:**
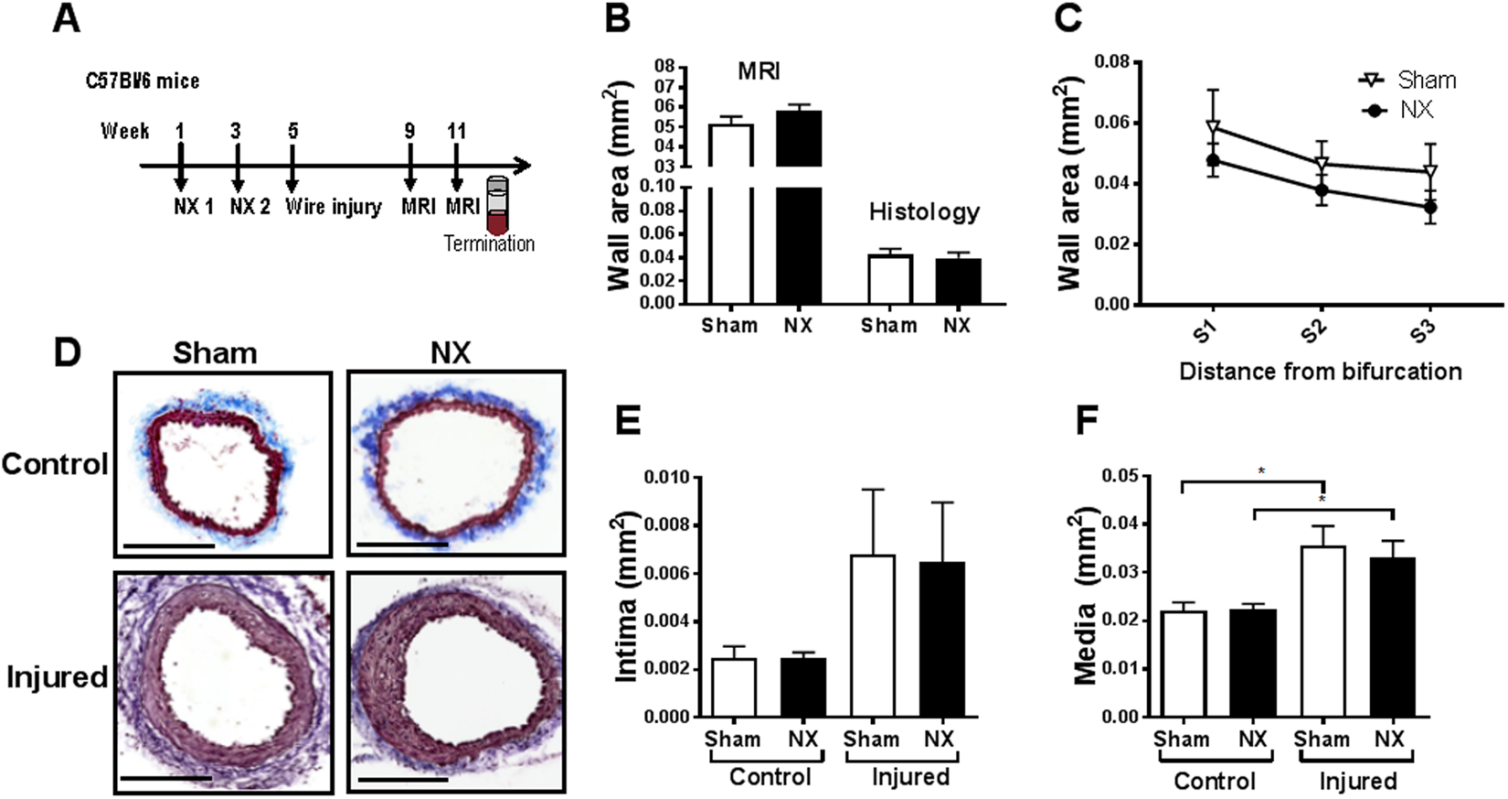
Uremia does not affect neointima formation in carotid arteries after vascular injury in C57BL/6 mice. (**A**) Study outline: C57BL/6 mice underwent 5/6 nephrectomy (NX) or sham operations in two steps (timepoints NX1 and NX2), and subsequently carotid wire injury and magnetic resonance imaging (MRI). (**B**) Quantification of cross-sectional vascular wall area by *in vivo* MRI (average of 4 cross-sections/mouse; n = 5 NX and n = 4 sham) and histology (average of 8–12 sections/mouse; n = 11 NX and n = 8 sham) in injured carotids of NX and sham C57BL/6 mice 6 weeks after wire injury. (**C**) Quantification of carotid wall area by histology. Three anatomical locations, series 1–3 (S1–S3, 2–4 sections/mouse/per series, n = 11 NX and n = 8 sham), were quantified. (**D**) Representative pictures of Trichrome stained injured and control carotid arteries from NX and sham mice. Scale bar, 200 μm. (**E**) Quantification of cross-sectional intimal and (**F**) medial area in injured and control carotid arteries from NX and sham mice (average of 8–12 sections/mouse, n = 11 NX and n = 8 sham). Data are presented as mean ± SEM. Unpaired t-test (**B**) with correction for multiple comparisons (**C**), 1-way ANOVA followed by Sidak’s multible comparison test (**E**,**F**). *P < 0.05.

To investigate the effect of uremia on neointima formation in wild type C57BL/6 (wt) mice, uremia was induced by 5/6 nephrectomy (NX). Control mice were sham operated (Sham). One week after the operations, NX induced increased plasma urea (2.2 fold, p < 0.0001), phosphate (1.2 fold, p < 0.01), and cholesterol (1.2 fold, p < 0.01) compared to sham-operated controls (Table 1). Two weeks after induction of uremia, mice underwent carotid wire injury (WI) in the left carotid artery, and the right carotid artery served as a control (Fig. 1A).

**Table 1:** Basic characteristics of uremic (5/6 NX) and sham-operated (sham) mice.

	C57BL/6		*Apoe* ^−/−^ 1 week after NX		*Apoe* ^−/−^ 8 weeks after NX	
	Sham	5/6 NX	Sham	5/6 NX	Sham	5/6 NX
n	13	12	12	20	12	20
Body weight (g)	23.5 ± 0.5	23.3 ± 1.1	20.5 ± 0.4	21.2 ± 0.2	23.5 ± 0.6	23.2 ± 0.3
P-Urea (mM)	8.9 ± 0.4	19.2 ± 1.6****	9.8 ± 1.3	20.1 ± 1.7***	6.1 ± 03	17.4 ± 2.5**
P-Calcium (M)	2.3 ± 0.0	2.6 ± 0.0****	2.5 ± 0.1	2.8 ± 0.0*	2.3 ± 0.0	2.5 ± 0.0**
P-Phosphate (mM)	2.2 ± 0.1	2.7 ± 0.1**	1.9 ± 0.1	1.7 ± 0.1	2.2 ± 0.1	2.3 ± 0.1
P-Cholesterol (mM)	2.3 ± 0.1	2.7 ± 0.1**	10.3 ± 0.8	12.8 ± 1.1	25.8 ± 1.5	32.3 ± 1.8*
P-Osteopontin (ng/ml)	—	—	—	—	264.5 ± 30	643.4 ± 63***

Values represent mean ± SEM. *p < 0.05; **p < 0.01; ***p < 0.001; ****p < 0.0001; Sham vs 5/6 NX, t-test.

**Figure 2:**
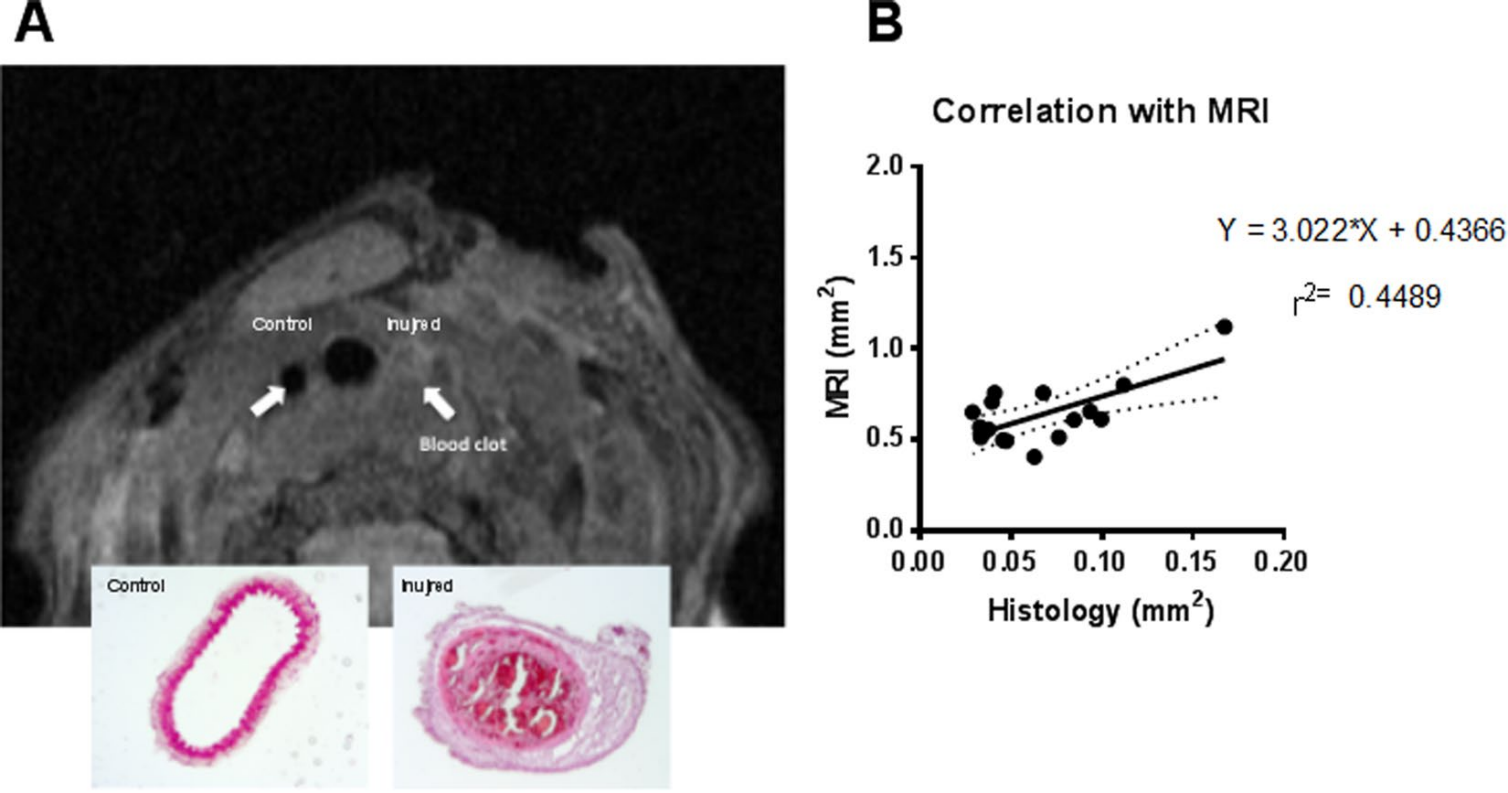
Magnetic resonance imaging on carotid arteries. (**A**) *In vivo* magnetic resonance imaging (MRI) and H&E staining from a C57BL/6 mouse subjected to wire injury in the left carotid artery. Following wire injury, the right carotid artery (control) appeared normal, whereas a blood clot obstructed the entire left (injured) carotid artery. (**B**) Correlation between MRI measurements and histological measurements of cross-sectional areas of injured carotid arteries. Values are averaged from 4 MRI cross-sections or 6–10 histological sections. n = 18. A statistically significant (p = 0.0045) correlation between MRI measured values and histology-derived values was found.

We had previously performed a pilot study comparing wall thickness measurements obtained using either magnetic resonance imaging (MRI) or histology in non-uremic wt mice. These analyses suggested that MRI can be used to compare neointima formation *in vivo* between groups of mice (Fig. 2B). Mice with blood clots in the carotids as judged by MRI were excluded (representative picture in Fig. 2A).

The carotid wall area was similar in NX and sham mice as measured by MRI four weeks after WI (Suppl. Fig. 1A). Based on this observation, the study was continued for two weeks. A significant difference in wall area between injured and control carotids was detected six weeks after WI (Suppl. Fig. 1B). However, there was no difference in the carotid wall area between NX and sham mice as determined by MRI and histology (Fig. 1B). This lack of effect of uremia was observed at three different anatomical locations (Fig. 1C). Also, we observed no difference in carotid medial or intimal areas between sham and NX mice (Fig. 1E,F). Based on immunohistochemistry, the content of $\alpha$ -SMA positive SMCs in injured carotid sections of NX and sham mice was similar (Suppl. Fig. 2A, top). There were no macrophages in the lesions as judged by MOMA-2 staining (Suppl. Fig. 2B, top).

**Figure 3:**
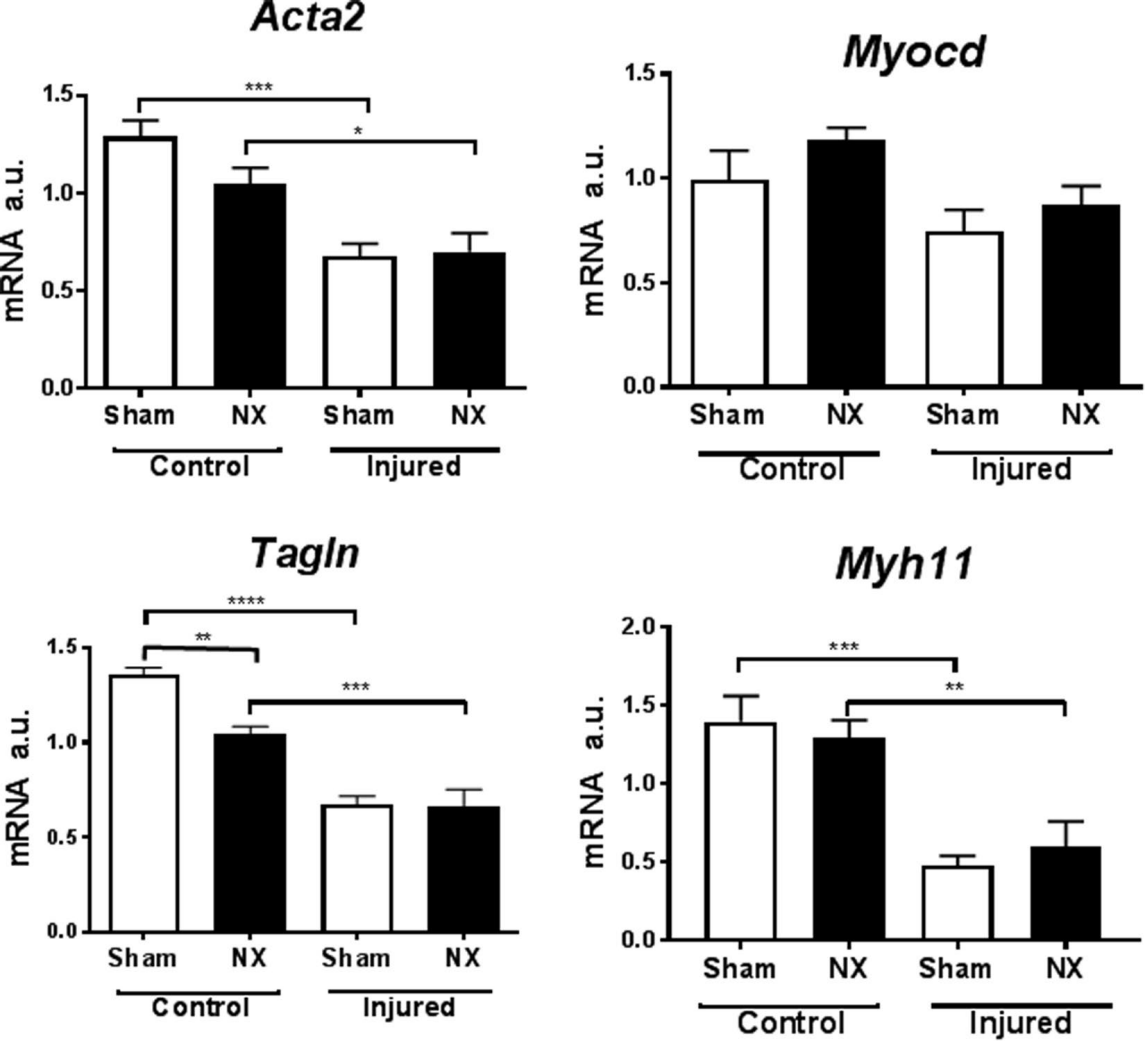
Wire injury, but not uremia, affects expression of smooth muscle marker genes in C57BL/6 mice. Real-time PCR analysis of carotid mRNA (pooled two and two) from sham-operated (sham) and uremic (NX) C57BL/6 mice, either with (Injured) or without (Control) wire-induced vascular injury. Gene expression was normalized to CANX. Bars indicate mean ± SEM (n = 6 per group); 1-way ANOVA followed by Sidak’s multible comparison test, *p < 0.05, **p < 0.1, ***p < 0.001,****p < 0.0001. a.u.: arbitrary units; *Acta2*: Smooth muscle alpha actin; *Myocd*, Myocardin; *Tagln*, SM22 alpha; *Myh11*, Smooth muscle myosin heavy chain.

As expected, mRNA markers of the contractile SMC phenotype were reduced in injured compared to control carotids (Fig. 3). Apart from *Tagln* mRNA expression, which displayed a statistically significant reduction in the uninjured control carotids of NX mice, NX did not affect mRNA expression of the SMC genes in control or injured carotids (Fig. 3).

### Uremia does not affect neointima formation in *Apoe*^−/−^ mice

**Figure 4:**
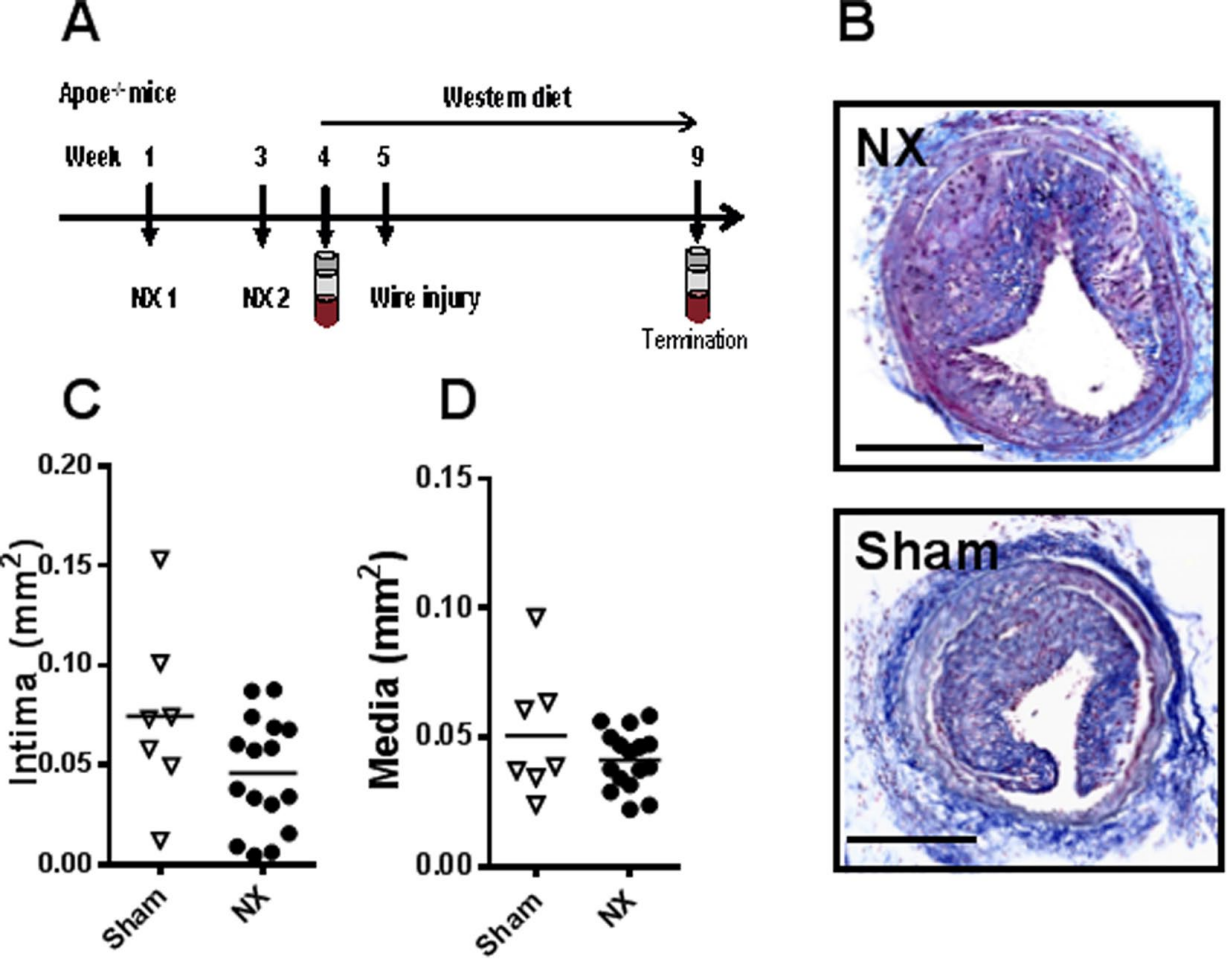
Uremia does not affect neointima formation in carotid arteries after vascular injury in *Apoe* ^−/−^ mice. (**A**) Study outline: *Apoe* ^−/−^ mice underwent 5/6 nephrectomy (NX) or sham operation (sham) in two steps (timepoints NX1 and NX2), were put on a western diet, and subsequently underwent carotid wire injury. **B**. Representative pictures of Trichrome stained injured carotid arteries from NX and sham *Apoe* ^−/−^ mice. Scale bar, 200 μm. (**C**) Histological quantification of cross-sectional intimal and (**D**) medial area in injured carotid arteries from NX and sham *Apoe* ^−/−^ mice (average of 8–12 sections per mouse; n = 16 NX, n = 7 sham), four weeks after wire injury. Horizontal bars represent mean values. Upaired t-test.

To address whether hypercholesterolemia potentiates neointima formation in itself, and potential effects of uremia on neointima formation, we performed a WI study in *Apoe* ^−/−^ mice (Fig. 4A). NX increased plasma urea (2 fold, p < 0.0001), and calcium (p < 0.05); there was no effect on plasma phosphate or cholesterol (Table 1). One week after induction of uremia, mice were shifted to a western-type diet for the remainder of the study. Two weeks after induction of uremia, all mice underwent carotid WI. Four weeks later (eight weeks after NX), NX mice had a 2.2 fold increase in plasma urea (p < 0.0001) compared to sham mice. Also, calcium and cholesterol concentrations were significantly increased in NX mice (Table 1). As anticipated, the carotid lesions were substantially larger in *Apoe* ^−/−^ than in wt mice (Fig. 1D and Fig. 4B). However, NX did not affect development of vascular lesions after WI in *Apoe* ^−/−^ mice (Fig. 4C,D). This lack of effect of NX was seen at four different anatomical locations in the injured area of the carotid artery (Suppl. Fig. 3A and B).

Based on histology, the composition of the vascular lesions in terms of macrophage, SMC, and collagen content was similar in carotid lesions from NX and sham mice (Suppl. Fig. 2A and B, bottom, depicts immunohistochemical stainings).

**Figure 5:**
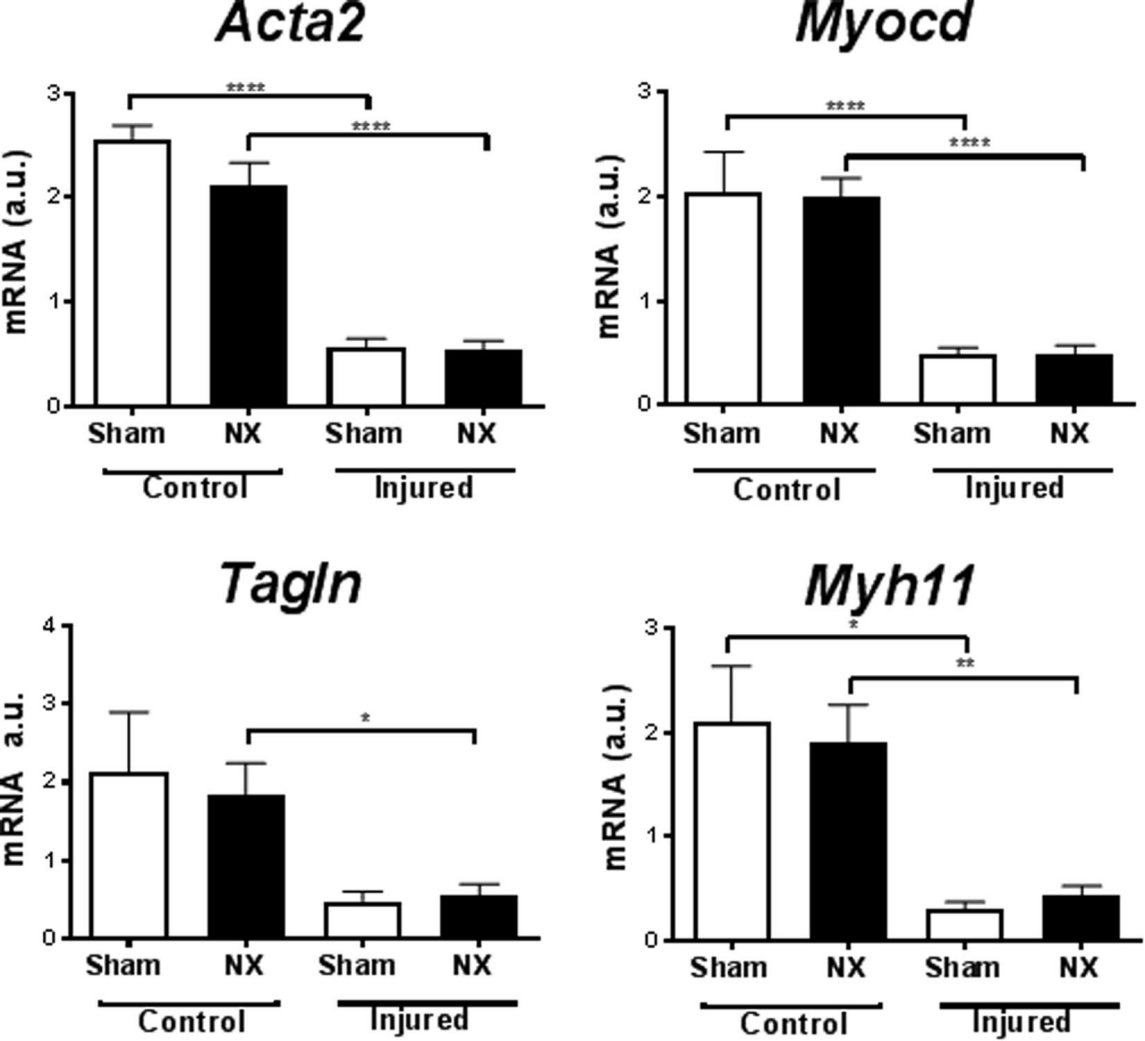
Wire injury, but not uremia, affects expression of smooth muscle marker genes in *Apoe* ^−/−^ mice. Real-time PCR analysis of carotid RNA (pooled two and two) from sham-operated (sham, n = 5) and uremic (NX, n = 10) *Apoe* ^−/−^ mice with (Injured) or without (Control) wire-induced vascular injury. Gene expression was normalized to CANX. Bars indicate mean ± SEM. 1-way ANOVA followed by Sidak’s multible comparison test, *p < 0.05, **p < 0.1, ***p < 0.001,****p < 0.0001. a.u.: arbitrary units. *Acta2*: Smooth muscle alpha actin; *Myocd*: Myocardin; *Tagln*: SM22 alpha; *Myh11*: Smooth muscle myosin heavy chain.

As in wt mice, the expression of mRNA markers of the contractile SMC phenotype was downregulated in injured compared to control carotids (*Acta2*, *Myocd*, *Tagln and Myh11*), and expression levels were similar in injured carotids from sham and NX *Apoe* ^−/−^ mice (Fig. 5). Furthermore, we assessed the expression of markers of endothelial cells, macrophages, and inflammation (platelet and endothelial adhesion molecule 1 (*Pecam1*), von Willebrand factor, interleukin-6, tumor necrosis factor α, F4/80, C-C chemokine receptor type 7). NX did not affect mRNA expression of any of these markers (data not shown). *Pecam1* expression was reduced in injured as compared to control carotid arteries (Suppl. Fig. 4).

## Discussion

This study aimed to assess whether uremia increases neointima formation in normocholesterolemic wt mice and hypercholesterolemic *Apoe* ^−/−^ mice by phenotypically modulating SMCs. The present evidence from histological and MRI analyses suggest that uremia affects neither the size nor the composition of the intimal and medial areas of injured carotid arteries. The morphological findings were further supported by gene expression analyses, since the expression of SMC marker genes were similar in NX and sham carotid arteries. Likewise, mRNA expression of endothelial, macrophage, and inflammatory markers was not affected by NX in the carotid artery. WI did induce a reduction in mRNA levels of *Pecam1*. At a first glance, this observation might signify lack of re-endothelialization. However, it might also merely be caused by the increased cellularity of injured carotid arteries, with a smaller fraction of endothelial cells compared to uninjured arteries.

Several previous observations supported the hypothesis that uremia would potentially increase neotintima formation by modulating the SMC phenotype. We have observed that *Myocd* and its target genes are downregulated in uremic aortas^25^. Reduction of *Myocd* is also seen in carotid arteries following wire injury, and viral overexpression of *Myocd* after carotid wire injury reduces neointima formation in mice^20^. Furthermore, overexpression of mir146a, which is increased in aortas of uremic mice^25^, promotes intimal hyperplasia in rats^27^. Also, vascular neointima formation is accelerated in arteriovenous fistulas in uremic rodents^28, 29^. However, as evident from the present study, uremia does not increase neointima formation in wt or *Apoe* ^−/−^ mice after carotid wire injury.

What is the explanation for these negative findings? Gene expression analyses in both wt and *Apoe* ^−/−^ mice indicate that wire injury in itself leads to phenotypic modulation of SMCs. As such, *Myocd*, *Acta2*, *Tagln*, and *Myh11* were downregulated in injured *vs* control arteries. Also, neointima formation was induced upon wire injury. To our surprise, the gene expression analyses showed no uremia-mediated effects on expression of the SMC genes in the carotid control arteries in wt or *Apoe* ^−/−^ mice (apart from *Tagln*, which was downregulated by NX in control arteries in wt mice). This is in contrast to previous data in aortic SMCs ^16, 18, 25, 26^, in which uremia altered mRNA expression of classical SMC marker genes. The gene expression differences observed in the previous *vs* the present study may thus relate to the different vascular beds examined, i.e. carotid artery in the present study *vs* aorta/*in vitro* data in previous studies. Indeed, the embryonic origin of SMCs is not identical in all segments of the vascular tree, and SMCs with different embryonic origin might respond differently to the same stimuli^30^.

It should be noted that the mice in our study are only mildly-moderately uremic. Hence, this uremic phenotype may not correspond to the phenotype observed in patients suffering from end-stage renal disease, which are at extreme risk of dying from CVD^5^. Furthermore, we cannot rule out the possibility that uremia-mediated effects on neointima formation could have been observed if mice had been uremic for a longer period of time. However, previous observations suggest that a mere two weeks of uremia increase systemic inflammation and phenotypic modulation of aortic SMCs^24^, both of which are key components in neointima formation after carotid wire injury. Thus, the six-seven weeks of uremia in the present study should be ‘sufficiently long’ to detect effects on carotid neointima formation.

In conclusion, our study show that uremia does not affect neointima formation in response to carotid wire injury in wt and *Apoe* ^−/−^ mice, possibly because uremia does not affect phenotypic modulation of SMCs in this vascular bed.

## Materials and Methods

### Mice

Female C57BL/6 (C57BL/6Ntac) and *Apoe* ^−/−^ (C57BL7&Jbom-Apoe^tm1Unc^) mice (Taconic, Ejby, Denmark) were housed under a 12 hour light/dark cycle in a temperature controlled room (21–23 °C) with free access to standard chow or western diet (D12079B, Research Diets Inc.) as indicated in the text. Animal experiments were performed according to the principles stated in the Danish law on animal experiments and were approved by the Animal Experiment’s Inspectorate, Ministry of Environment and Food, Denmark (permission no. 2013-15-2934-00773). The investigation conforms to the Guide for the Care and Use of Laboratory Animals published by the European Parliament [EU directive 2010/63/EU].

Surgeries were performed under anesthesia with Zoletil (Tiletamin 1,63 mg/mL, Zolazepam 1,63 mg/mL, Xylazin, 2,61 mg/mL, Butorphanoltartrat 0,065 mg/mL) at a dose of 0.01 mL/g body weight. Analgesia (buprenorphine 0.001 mg/10 g body weight) was administered subcutaneously twice daily for up to three days after the surgical procedures.

### 5/6 Nephrectomy

Uremia was induced by 5/6 nephrectomy (NX) in a two-step surgical procedure as previously described^15, 31^. Briefly, both poles of the right kidney were resected leaving an intact kidney segment, and two weeks later the entire left kidney was removed. Control mice underwent sham-operations exposing first the right kidney and two weeks later the left kidney. After kidney manipulation or exposure, the peritoneum was sutured with 4-0 sutures and the skin incision with 3-0 sutures.

### Carotid artery wire injury

All mice underwent transluminal wire injury of the left common carotid artery (CCA) with a 0.36 mm flexible guide wire (Hi-Torque balance middleweight, Abbot, 1001780-HC). The left CCA was exposed via a midline incision at the ventral side and the bifurcation located. Silk ligatures (6-0, PERMA-HAND, Ethicon) were placed around the external carotid artery and distally around the CCA. Two sutures were placed around the internal carotid artery, which was then tied off with the distal ligature. An incision hole was made between the two ligatures and the guidewire was introduced via the incision hole into the CCA. Denudation was achieved by 3 passes along the vessel and 3 rotational passes. The proximal ligature was tied off and blood flow restored. The skin incision was closed using 4-0 sutures.

Animals were euthanized 4–6 weeks after wire injury. Mice were anaesthetized with Zoletil (0.01 mL/g), bled from the orbital plexus and perfused with cold saline. Carotids were cut in two and the upper section was divided into two pieces. The upper piece was fixed in 4% formaldehyde for 30 minutes, transferred to cryoembedding media (Tissue-Tek® O.C.T.) and frozen. The lower part was snap frozen for RNA analysis.

### Plasma measurements

Blood was collected in heparinized microtubes and centrifuged at 4000 rpm for 10 min at 4 °C. Plasma concentrations of urea, creatinine, phosphate, and cholesterol were measured on a Cobas® 8000 modular analyzer series (Roche A/S). Plasma concentrations of osteopontin were measured with the quantikine mouse osteopontin immunoassay (MOST00) according to the manufacturer’s instructions (R&D Systems, Abingdon, UK).

### MRI

Anatomical MR images were acquired on a BioSpec 7 T/16 cm system (Bruker) running Paravision 5.1 (Bruker). T2-weighted proton density scan was obtained using a multi-slice multi-echo sequence (MSME-PD-T2) and a 30 mm surface RF coil. The following parameters were used: TR = 2000 ms, TE 13/65 ms, 1 average, 10 slices, slice thickness 0.60 mm, inter slice distance 0.60 mm, in plane pixel size 97.7 × 97.7 µm. Images were acquired in the axial plane. Image analysis was performed in Horos v2.0.1. The area of the carotid artery wall was estimated by drawing region of interests (ROIs) covering the outer artery wall and subtracting the area for ROIs drawn over the lumen.

### Histology

Starting at the bifurcation, 10 um thick sections were collected in series of 5 glass slides (SuperFrost Plus slides, Menzel-Glaser, Germany) with 4 sections per glass. For wt mice, each series was separated by 100 um. For morphometry, 1 glas from 3–4 subsequent series were stained with Masson’s trichrome (Sigma-Aldrich, HT15-1KT) according to the manufactures instructions. Intimal and medial areas were quantified and the collagen-positive area was determined as the percentage of blue-staining using the image analyses software Visiopharm (Visiopharm, Hørsholm, Denmark).

To stain macrophages, we performed anti-MOMA-2 immunohistochemistry as previously described^32^ with the following modifications: Sections were incubated with primary MOMA-2 antibody (rat anti-mouse IgG2b, Serotec, Düsseldorf, Germany, MCA519) diluted 1:300 overnight at 4 °C.

To stain smooth muscle cells, a biotinylated primary antibody (Actin, smooth Ab (clone 1A4); MS-113- B1; Neomarkers, Freemont, CA, USA) was applied. Endogenous peroxidase activity was blocked with 0.5% H_2_O_2_, rinsed in dH_2_O and antigen retrieval was obtained with proteinase K treatment for 2 minutes and rinsed in TBS-T, and unspecific binding was blocked with 2% BSA. Sections were incubated with primary antibody (0.2 ug/ml in 1% BSA in TBS) over night at 4 °C. After rinse in TBS-T, antibody binding was detected with the Vectastain, Elite ABC kit (VWR, Denmark, PK6100) and DAB (Dako), and sections were counterstained with Mayer’s haematoxylin. MOMA-2 and $\alpha$-SMA staining was scored independently by two investigators from 0–3 based on area and intensity.

For CD31 staining, endogenous peroxidase was blocked with 0.5% H_2_O_2_, antigen retrieval was obtained with proteinase K treatment, and sections were blocked with 2% BSA in TBS-T for 1 hour. Sections were incubated with primary anti-Cd31 antibody (ab28364, Abcam; 0.5 ug/ml) over night at 4 °C, and the EnVision System HRP-labeled Polymer anti-rabbit (Dako, K4002) followed by DAB (Dako) was used for detection.

### RNA extraction and qPCR analyses

To obtain enough RNA for subsequent analyses, carotid samples were pooled two and two, the tissue was homogenized on a Tissuelyzer, and RNA was extracted using a mirRNeasy Micro Kit (Qiagen, 217084E). RNA quality was assessed on a BioAnalyzer (Agilent Technologies Denmark A/S, Naerum, Denmark) using an RNA 6000 Nano Assay Kit and RNA concentration was determined on nanodrop. For mRNA analyses, cDNA was made from 54–75ng RNA with High Capacity cDNA Reverse Transcription Kit (Roche, Applied biosystems, 4368814) in a 10 μl reaction at 37 °C for 120 minutes. Real time PCR was performed on an ABI Prism 7900HT Sequence Detection System. Each reaction mixture contained 1.08–1.5 ng cDNA and the ABI Fast SYBR Green Mix or TagMan®Gene Expression master mix (Applied Biosystems) was used. Standard curves were made by serial dilution of a pool of all cDNAs. All analyses were done in duplicate, and gene expression data were normalized against expression of CANX and ACTB (Primer/Double Dye, DD-mo-600, Primerdesign). Primer sequences are listed in Suppl. Table 1.

### Statistics

Data were analyzed using the Graphpad Prism software, version 7 (GraphPad Inc.). P values were calculated using one-way ANOVA following Sidak’s post-hoc test, or unpaired t-test as indicated in the manuscript. Correction for multiple comparisons was done by the Holm-Sidak test. p < 0.05 was considered significant. Values represent mean ± SEM.

### Data availability

The datasets generated during and/or analysed during the current study are available from the corresponding author on reasonable request.

## Electronic supplementary material

Supplementary Information
