# Supplementary material for: Uremia does not affect neointima formation in mice
Source: Sci Rep. 2017 Jul 26;7:6496. doi: 10.1038/s41598-017-06816-6 (PMC5529519; doi:10.1038/s41598-017-06816-6)
Supplement: Supplementary file 1 — Supplementary Information [file 41598_2017_6816_MOESM1_ESM.pdf]

Supplementary information

Uremia does not affect neointima formation in mice

Annemarie Aarup[1], Carsten H. Nielsen[2], Line S. Bisgaard[1], Ilze Bot[3], Henrik H. El-Ali[2], Andreas Kjaer[2], Lars B. Nielsen[1,4], and Tanja X. Pedersen[1]

[1] Department of Biomedical Sciences, University of Copenhagen, Copenhagen, Denmark

[2] Department of Clinical Physiology, Nuclear Medicine & PET and Cluster for Molecular Imaging, Rigshospitalet, University of Copenhagen, Copenhagen, Denmark

[3] Division of Biopharmaceutics, Leiden Academic Centre for Drug Research, Leiden University, Leiden, The Netherlands

[4] Copenhagen University Hospital, Rigshospitalet, Department of Clinical Biochemistry (Copenhagen, (Denmark)

Corresponding author: Tanja X. Pedersen (tanjax@sund.ku.dk)

Suppl. Figure 1

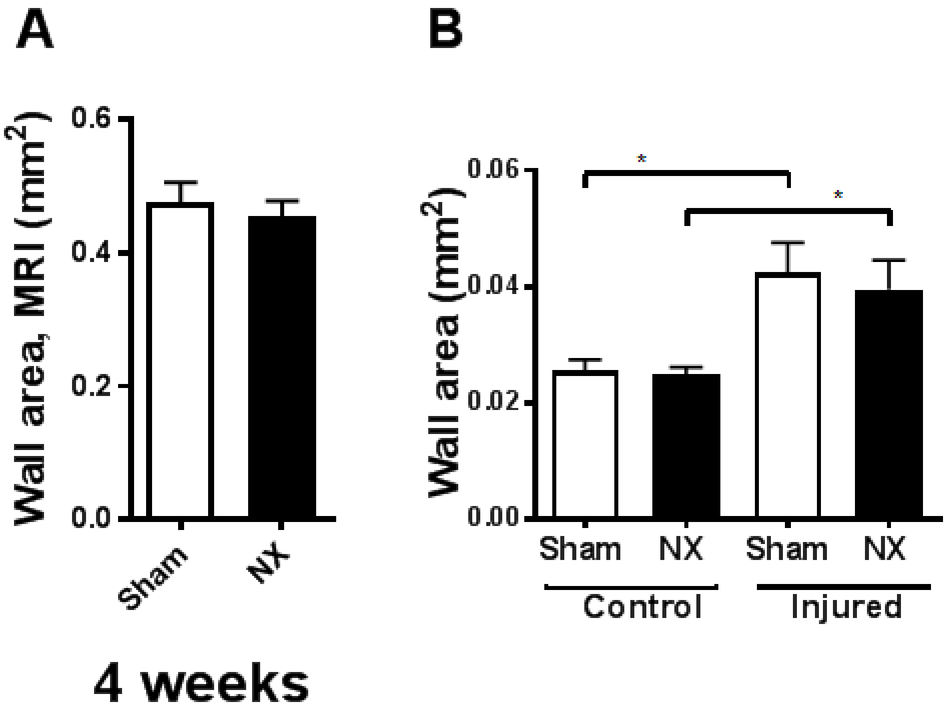

Suppl. Figure 1. Uremia does not affect carotid wall area following wire-induced injury.
A. Quantification of cross-sectional vascular wall area by in vivo serial magnetic resonance imaging (MRI) in injured carotids of uremic (NX) and sham-operated (sham) C57Bl/6 mice 4 weeks after wire injury (average of 4 serial pictures per mouse, n=5/group). **B**. Quantification of cross-sectional vascular wall area of injured and control carotid arteries from NX (n=11) and sham (n=8) mice. Average of 8-12 sections per mouse. Data are presented as mean±SEM. *$p<0.05$. Unpaired t-test (**A**) or 1-way ANOVA followed by Sidak's multible comparison test (**B**).

Suppl. Figure 2

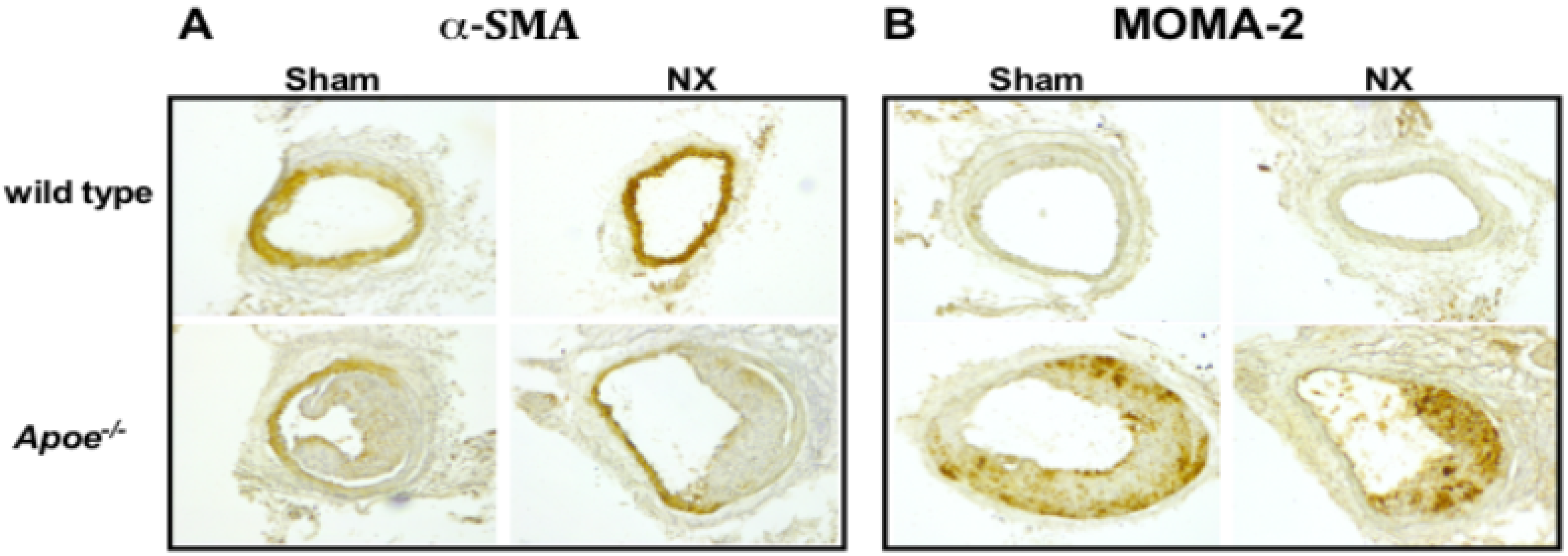

Suppl. Fig 2. Uremia does not affect smooth muscle and macrophage staining in carotid arteries after vascular injury in wild type and Apoe-/- mice.
Immunohistochemical analysis of carotid arteries from sham-operated (sham) and uremic (NX) wild type and Apoe-/- mice following wire-induced vascular injury. **A**. Sections stained for the smooth muscle marker α-SMA. **B**. sections stained for the macrophage marker MOMA-2. Brown staining indicates positive cells in both **A** and **B**.

Suppl. Figure 3

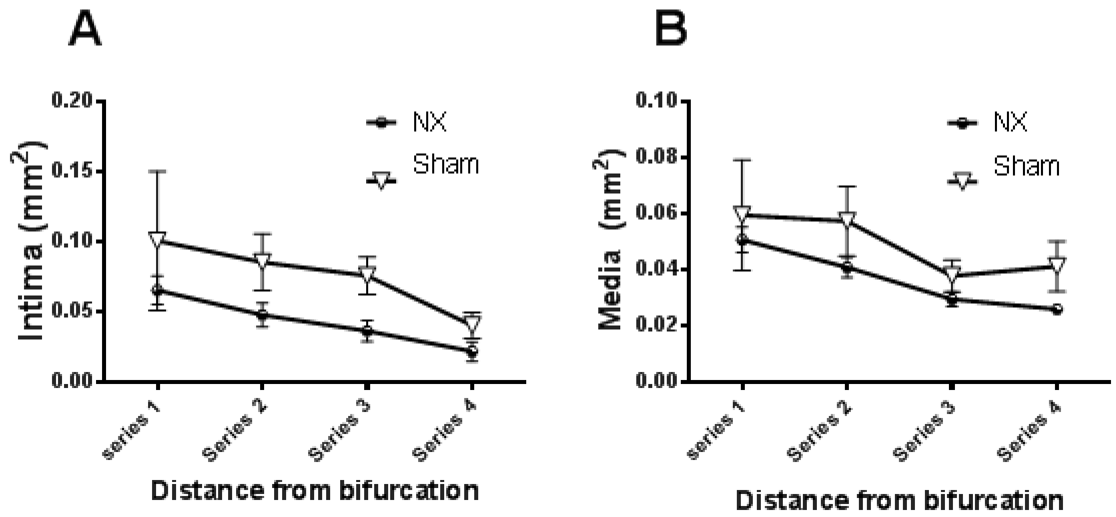

Suppl. Figure 3. Uremia does not affect the intimal or medial area after vascular injury in carotids of Apoe-/- mice.
Histological quantification of cross-sectional intimal (**A**) and medial (**B**) areas in injured carotid arteries from uremic (NX, n=11) and sham-operated (sham, n=7) Apoe-/- mice, 4 weeks after wire injury. Sections are from 4 different anatomical locations, series 1-4, average of 2-4 sections/mouse per series. Unpaired t-test with Holm-Sidak correction for multiple comparisons.

Suppl. Figure 4

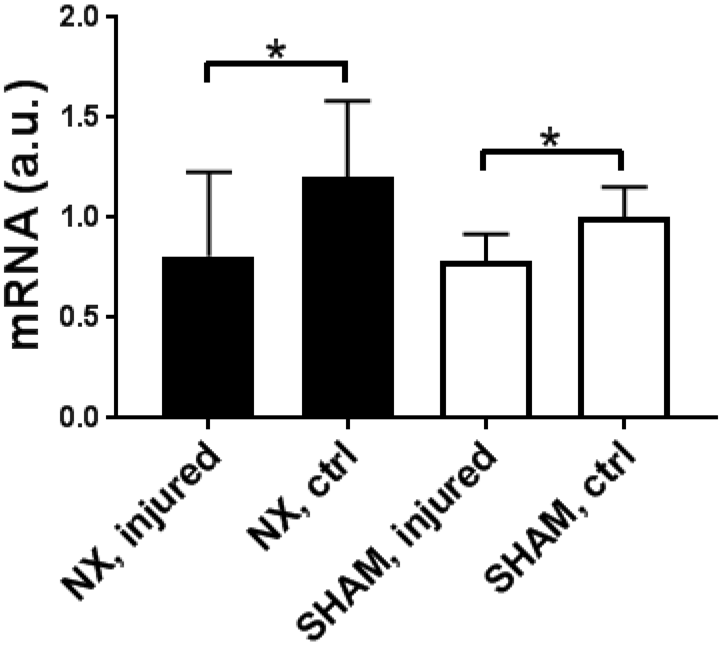

Suppl. Figure 4. Wire injury leads to downregulation of mRNA expression of PECAM1.
mRNA expression of PECAM1 in injured or un-injured control (ctrl) carotids from sham-operated (sham) or uremic (NX) mice. PECAM1 expression was normalized to expression of the housekeeping gene CANX and is reported in arbitrary units (a.u.). Depicted values are mean +/- SD. *p<0.05. Unpaired t-test.
